# Emergence of a novel zoonotic brugian filarial infection during post-validation surveillance for lymphatic filariasis in Sri Lanka

**DOI:** 10.1016/j.lansea.2026.100735

**Published:** 2026-03-04

**Authors:** Indeewarie E. Gunaratna, Lucas G. Huggins, Ushani Atapattu, Lakmini Liyanage, Nipuni Shilpeswarage, Murali Vallipuranathan, Vito Colella

**Affiliations:** a Anti-filariasis Campaign, Ministry of Health, Colombo 5, Sri Lanka; b Melbourne Veterinary School, Faculty of Science, University of Melbourne, VIC, 3010, Australia

**Keywords:** Neglected tropical diseases, *Brugia*, Lymphatic filariasis, Zoonosis, Molecular sequencing

## Abstract

**Background:**

Lymphatic filariasis (LF) affects approximately 120 million people globally and is caused by *Wuchereria bancrofti*, *Brugia malayi* and *Brugia timori*. Sri Lanka was historically endemic for both bancroftian and brugian filariasis, with *B. malayi* presumed eliminated by the late 1960s. The country achieved validation of LF elimination as a public health problem in 2016. However, sporadic *Brugia* infections have been detected in microfilaria surveys from the mid-2000s onwards; the sub-periodic periodicity of which is suggestive of a zoonotic origin and warrants further research.

**Methods:**

Night blood survey (NBS) surveillance was conducted by the Anti-Filariasis Campaign, Ministry of Health, Sri Lanka, from 2019 to 2023. *Brugia*-positive human samples and archived canine samples were analysed using a next-generation sequencing metabarcoding platform targeting mitochondrial and nuclear loci. Bayesian and neighbour-joining phylogenetic analyses and minimum-spanning network approaches were used to characterise lineage relationships.

**Findings:**

Among 1,855,165 individuals screened, 52 were identified with *Brugia*-like microfilariae. Metabarcoding generated 1.2 million high-quality reads with both mitochondrial *cox1* and ribosomal DNA markers confirming a single *Brugia* lineage circulating in humans, mainly in children. Sequences of the *cox1* gene showed 99.4–100 percent nucleotide identity to the *Brugia* Sri Lanka (SL) genotype previously detected in dogs in Sri Lanka and Tamil Nadu (India). rDNA sequences showed low similarity to *B. malayi* or *Brugia pahangi*, indicating that the parasite represents a distinct *Brugia* taxon lacking a reference rDNA sequence.

**Interpretation:**

The findings provide evidence that human brugian filariasis in Sri Lanka is caused by a previously unrecognised zoonotic *Brugia* species maintained in dogs. This has important clinical and public health implications given that human infections have occurred in children despite LF being declared eliminated as a public health problem. Crucially, the incorrect classification of circulating filarioid species may lead to inappropriate clinical and programmatic responses. For example, the newly identified canine reservoir of this *Brugia* genotype means that human-only mass drug administration might not be sufficient to interrupt this species’ transmission. These results underscore the need for integrated One Health surveillance to prevent re-establishment of LF and safeguard regional elimination goals.

**Funding:**

No specific funding was received for the present study. This study was supported by a consulting funding (PRJ_002971).

**Research in context**

**Evidence before this study**

We searched PubMed for English-language studies from inception to October 10th, 2025, for studies reporting on the emergence of human filariasis caused by *Brugia* species with a zoonotic origin using the search term ‘Sri Lanka AND Brugia AND zoonotic AND emerging human filariasis’ with relevant synonyms. A total of five studies were returned and whilst two studies reported on the characterisation of a novel *Brugia* species in dogs, none described the detection or characterisation of *Brugia* infecting people in Sri Lanka.

**Added value of this study**

Human blood samples that were found to be *Brugia* positive through regular night blood smear surveillance in Sri Lanka were genetically characterised at two genomic loci using an advanced sequencing platform. Through this characterisation, a single *Brugia* genotype was identified as circulating in humans, mainly children, which is genetically identical to a genotype detected in canines from Sri Lanka and the south of India. This *Brugia* genotype is genetically distinct from *Brugia malayi* which is the most closely related species as inferred through phylogenetic analyses.

**Implications of all the available evidence**

This study provides evidence that a novel *Brugia* species that infects canines also causes re-emerging human brugian filariasis in Sri Lanka, using these animals as a reservoir host. Given the potential for dog-to-human transmission, the re-emergence of brugian filariasis since the cessation of mass drug administration in Sri Lanka has critical implications for global lymphatic filariasis elimination efforts and underscores the need for approaches that consider zoonotic reservoirs to control this devastating neglected tropical disease.

## Introduction

Lymphatic filariasis (LF) is recognised as a neglected tropical disease (NTD) by the World Health Organisation (WHO), and affects over 120 million individuals, predominantly in low and lower-middle-income countries.^1^ The filarial worm, *Wuchereria bancrofti*, causes the majority of these infections, whilst *B. malayi* and, to a lesser extent *Brugia timori*, contributes to the remaining 10% of infections.^1^ Lymphatic filariasis, when progressed, leads to a chronic, debilitating and socially stigmatising disease through the blockage and impairment of the correct functioning of the lymphatic system that significantly impacts the quality of life for affected individuals.^2^

Sri Lanka has been endemic for LF since the 12th century AD, with brugian filariasis (caused by a species identified as *B. malayi* at the time) recognised as the primary cause of this disease prior to World War II.^3^ Reports from this period highlight clinical signs consistent with LF, such as elephantiasis and “pain in the legs” in regions endemic for brugian filariasis.^3^ Subsequently, LF caused by *Brugia* spp. was displaced by *W. bancrofti* in the aftermath of the war, and by the late 1960s, brugian filariasis was no longer reported from Sri Lanka, potentially signalling its elimination from the country.^3^ Importantly, with the aim of eliminating bancroftian filariasis, Sri Lanka launched a mass drug administration (MDA) campaign under the WHO's Global Program to Eliminate Lymphatic Filariasis (GPELF) between 2002 and 2006. Finally, in 2016 Sri Lanka was designated as having eliminated LF as a public health problem.^4^

Despite the commendable successes of the filariasis control campaign in Sri Lanka, patients with *Brugia* infections, identified as *B. malayi*, have recently been detected during the post-validation surveillance phase, marking the first human cases of brugian filariasis since the 1960s.^5^, ^6^, ^7^ Such findings pose a risk to Sri Lanka's LF elimination status and underscore the possibility that brugian filariasis may have been underreported. These re-emergent *Brugia* infections were found to be sub-periodic in contrast to nocturnally periodic infections that had predominated in the past.^8^ Sub-periodic *Brugia* is known to be associated with potentially zoonotic reservoirs,^9^ which had led to the suspicion that an animal reservoir may be contributing to the recent increase in human *Brugia* infections in Sri Lanka.^8^

Several studies have reported the presence of *Brugia* in dogs and cats in Sri Lanka.^10^, ^11^, ^12^, ^13^, ^14^ Most of these have identified the *Brugia* species infecting dogs as *B. malayi* through the use of microscopic techniques^12^ or molecular diagnostics that use barcoding gene targets that are suboptimal to identify these parasites at a species level.^13^ Recently, a comprehensive molecular analysis using an appropriate barcoding target, specifically the mitochondrial cytochrome oxidase I (*cox1*) gene, has revealed that *Brugia*-infecting dogs in Sri Lanka is molecularly distinct from the *B. malayi*, found across Southeast Asia.^14^ This distinct strain has been temporarily identified as *Brugia* sp. Sri Lanka genotype (*Brugia* SL).^14^ At the same time, *Brugia ceylonensis*, another *Brugia* species historically reported from Sri Lanka, has not been reported since its first description in 1962, a time when investigations were solely conducted through microscopic and morphological analysis. The absence of molecular data for *B. ceylonensis* is a significant challenge when attempting to compare modern day *Brugia* SL to historical reports of *B. ceylonensis.* Nonetheless*, B. ceylonensis* may be the likely aetiology of the re-emergent *Brugia* in the country.^7^^,^^14^

*Brugia* SL has been identified in dogs at a prevalence of nearly 20% in regions that are endemic for LF in humans in Sri Lanka compared to nearly 1.5% prevalence in regions that are non-endemic for LF.^14^ This suggests that dogs may be playing a role as reservoirs for these re-emergent *Brugia* infections in humans.^12^ However, empirical evidence to support this hypothesis is absent, thereby adversely impacting LF control in Sri Lanka.

Here, we performed a comprehensive molecular analysis on microfilaraemic blood samples containing *Brugia* spp. microfilariae from humans in Sri Lanka using a novel pan-filarial worm metabarcoding diagnostic tool.^15^ With the aid of this tool, we provide evidence that *Brugia* SL genotype is endemic in humans in Sri Lanka and that dogs are reservoirs for re-emergent brugian filariasis in the country with implication for national control strategies.

## Methods

**Fig. 1:**
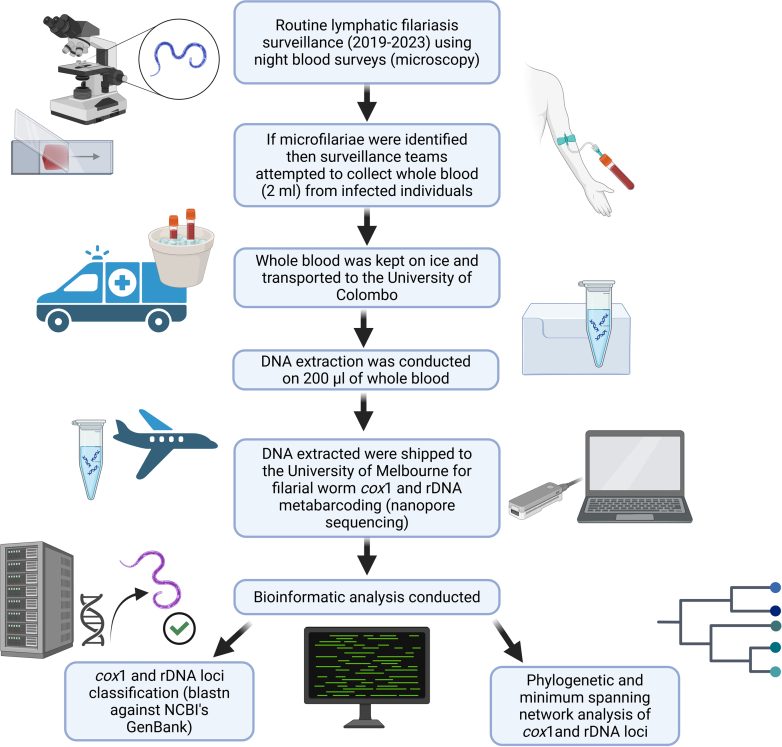
**Flow chart of field work and collection procedures, laboratory work and bioinformatic analysis pipelines used for this study**. Created in https://BioRender.com.

Routine active surveillance for LF using night blood surveys (NBS) was conducted by trained teams from the Anti-Filariasis Campaign (AFC), Ministry of Health, Sri Lanka between 2019 and 2023 from LF endemic provinces, specifically in the districts of Puttalam, Kurunegala, Gampaha, Colombo, Kalutara, Galle, Matara and Hambantota (Supplementary Figure S1). NBS were carried out by trained teams at the individual's residence according to approved standard operating procedures i.e. two thick blood smears followed by Giemsa staining per patient.^16^ If sheathed microfilariae with two terminal nuclei resembling those of *B. malayi* were detected,^14^^,^^17^ then 2 ml blood samples would be attempted to be collected in ethylenediaminetetraacetic acid (EDTA) tubes by a trained team comprising a phlebotomist and a public health field officer at the individual's residence. Blood samples were transported to the AFC's laboratory on ice and were stored at 4 °C until further analysis. Patients found positive during the NBS were treated with diethylcarbamazine citrate 6 mg/kg/day for 12 days (total cumulative dose of 72 mg/kg) in combination with a single dose of albendazole (400 mg). A flow chart showing the complete fieldwork workflow as well as laboratory and bioinformatic analysis pipeline used for this study is shown in Fig. 1.

### DNA extraction

DNA from these stored blood samples was extracted from 200 μl starting material using the DNeasy Blood and Tissue Kit (Qiagen, Hilden, Germany) according to the manufacturer's protocol. The extracted DNA was then shipped to the Melbourne Veterinary School, University of Melbourne (Australia) on ice and stored at −20 °C upon arrival. Prior to further analysis, DNA was quantified using a Qubit™ 4 Fluorometer (Thermo Fisher Scientific, Massachusetts, USA) using the dsDNA HS assay kit (Thermo Fisher Scientific, Massachusetts, USA) to ascertain the quantity of DNA in each sample. Archived DNA from Sri Lankan canine blood samples, previously identified as positive for *Brugia* SL in an earlier study (*n* = 19),^14^ was quantified as described above and used in the analysis.

### Filarial worm metabarcoding

DNA extracted from human and canine blood from Sri Lanka was subjected to a pre-validated and published nanopore-based metabarcoding assay^15^ which amplifies and sequences the almost entire filarioid *cox1* gene to provide a species-level classification of all such pathogens present in the sample. A known *B. malayi*-positive sample from Thailand was also included to assess whether any errors were introduced by the sequencing technique used in this study. Library preparation was carried out exactly as per Huggins et al. (2024),^15^ utilising Oxford Nanopore Technologies' (ONT) PCR Barcoding Expansion 1–96 (EXP-PBC096) and Ligation Sequencing Kits (SQK-LSK110) with deep-sequencing on the MinION™ Mk1B device (Oxford Nanopore Technologies, Oxford, UK). The library prepared amplicons from all samples were pooled in equimolar quantities, underwent DNA repair, end-prep and adapter ligation and were finally loaded onto a new R9.4.1 flow cell at a concentration of 47.05 fmol. Filarial worm metabarcoding of the *cox1* gene for human samples was conducted using a separate sequencing library and flow cell run to those used for *cox1* metabarcoding on canine samples to obviate any potential issues with barcode crosstalk/barcoding hopping. This sequencing library was also run with two PCR negative controls (sterile water) and two positive controls as per the method detailed in Huggins et al. (2024).^15^ Positive controls were used to determine a read cut-off threshold to accurately determine whether a sample was truly positive for a given filarial worm, as defined in Huggins et al. (2024).^15^

A second metabarcoding assay was also developed and employed which targets the 3′ end of the 18S ribosomal RNA (18*S rRNA*) gene, the entire internal transcribed spacer 1 (*ITS*-1) region, the entire 5.8S ribosomal RNA (5.8*S rRNA*) gene, the entire internal transcribed spacer 2 (*ITS*-2) region and the 5′ end of the 28S ribosomal RNA (28*S rRNA*) gene. This genomic metabarcoding target is from here on referred to as the ribosomal DNA (rDNA) region. The successful sequencing of this large section of the nuclear ribosomal rDNA region of filarial worm species was designed to further support the taxonomic classification results provided by the mitochondrial *cox1* gene. In silico development of primers aimed at amplifying a large (>1000 bp) portion of the filarial worm rDNA region were developed using a multi-species filarioid genetic alignment in Geneious Prime® 2023.2.1 using standard primer design guidelines and the online tools Primer3web version 4.1.0 (https://primer3.ut.ee/) and OligoAnalyzer™ Tool (https://www.idtdna.com/pages/tools/oligoanalyzer). Laboratory-based *in vitro* testing of the six novel primers that were designed was conducted using an array of previously characterised filarial worm positive controls for *W. bancrofti*, *B. malayi* and *Brugia pahangi*. Primer performance was assessed based on the presence of observable bands when PCR product was visualised on 1.5% agarose gels dyed with GelRed® (Biotium, California, USA) using a ChemiDoc™ Imaging System (Bio-Rad, California, USA). Through this process, one primer pair: Fil_rRNAF13_ONT 5′-TTTCTGTTGGTGCTGATATTGCGCGCTACACTGGAGGAATCA-3′ and Fil28S_R1_ONT 5′-ACTTGCCTGTCGCTCTATCTTCGCTGCAATCTCAAACAACCCG-3′ which includes the required ONT adapter sequences (underlined) were found to perform best, i.e. produce a single clear and bright band on a gel, and amplify an approximately 1470 bp section of the nuclear rDNA region. The first step PCR thermocycling conditions with these primers were: 1 cycle of 94 °C for 30 s, 25 cycles of 94 °C for 20 s, 58 °C for 30 s and 65 °C for 90 s, with a final extension of 65 °C for 10 min. For the second step PCR through which unique indexes were added to each sample the thermocycling conditions followed those suggested by the relevant ONT protocol, specifically these were 1 cycle of 95 °C for 3 min, 12 cycles of 95 °C for 15 s, 62 °C for 15 s and 65 °C for 90 s, with a final extension of 65 °C for 5 min. The rest of the library preparation for the rDNA region amplicons was conducted as described for the *cox1* gene above.

Nanopore sequencing was conducted on a MinION Mk1B device using a Legion 7i Gen 6 laptop (Lenovo, Quarry Bay, Hong Kong) for 48 h, as defined in Huggins et al. (2024).^15^ POD5 data were then base-called in MinKNOW version 23.11.7 using the super high accuracy base-calling model with barcode removal using Dorado version 7.2.12.

All nanopore sequencing data produced in this study are available from the NCBI BioProject database BioProjectID: PRJNA1125935.

### Bioinformatic analysis of metabarcoding data

All bioinformatic processing was conducted using the NanoCLUST pipeline as it corrects for the error rate of nanopore flow cells by construction of accurate barcoding gene consensus sequences.^18^ After quality control assessment of FASTQ data using fastp^19^ the selected NanoCLUST parameters were taken from Huggins et al. (2024) for the *cox1* gene,^15^ whilst for the rDNA region analysis optimal parameters were a minimum read length of 1200 bp, maximum read length of 1800 bp, a HDBSCAN cluster selection epsilon value of 1, minimum cluster size of 30 and 30 reads used for polishing, with all other parameters kept as the pipeline's defaults. NanoCLUST was therefore used to generate each samples' consensus sequences for the filarial worm *cox1* gene and rDNA regions. Next, these consensus sequences were classified by comparing them both to an in-house filarial worm reference database for each target locus and to the full NCBI GenBank database using blastn.^20^

Read thresholds to determine whether a sample is positive for a given filarial worm were calculated using a pre-validated method.^15^^,^^21^ In brief, if uniquely identifiable positive control reads were found in samples other than the positive control then this read count was taken as the read cut-off threshold for the sequencing batch.

### Phylogenetic analysis and sequence type network construction

Available *Brugia* spp. mitochondrial *cox1* gene sequences as of 23.02.2024 greater than 400 bp were downloaded from the GenBank database and imported into Geneious Prime® 2023.2.1. Unique sequence types (STs) of these *Brugia* sequences were selected and aligned using the MAFFT algorithm, together with the representative sequences for humans and dogs obtained through the metabarcoding analysis in this study and from a pre-published study,^15^ respectively. The alignment was exported as a FASTA file and used to determine the best nucleotide model for phylogenetics through maximum likelihood analysis in MEGA Version 11.0.13.^22^ The model with the lowest Bayesian Information Criterion (BIC) value was chosen for phylogenetic inference.

Phylogenetic inference was performed with Bayesian and neighbour-joining (NJ) approaches. For Bayesian inference (BI), the FASTA alignment was converted to a NEXUS file containing a code block with instructions for BI with MrBayes 3.2.7^23^ using Mesquite: a modular system for evolutionary analysis (Version 3.70).^24^ Two million Markov Chain Monte Carlo (MCMC) generations were carried out, sampling every 100th generation and allowing for both transitions and transversions with gamma-distributed rates. A burn-in rate of 25% was employed. NJ analysis was performed in MEGA Version 11.0.13^22^ using 2000 bootstrap replicates with the Tamura-Nei model and gamma-distributed rates allowing for transitions and transversions. Both BI and NJ analyses parameters were set to perform analyses using the nucleotide model identified earlier.

The rDNA region sequences generated in this study, including those from previously characterised positive controls from dogs and humans in Sri Lanka, were used because rDNA sequences of comparable lengths were unavailable for most filarial pathogens in publicly accessible databases such as GenBank. These sequences were then imported into Geneious Prime® 2023.2.1 and used for phylogenetic inference using both Bayesian and Neighbour-Joining (NJ) approaches, similar to the analysis of *cox1* gene sequences described above. A Tamura 3-parameter model with gamma-distributed rates was applied, as it was identified as the best-fit model for the alignment.

A minimum-spanning sequence type (MS-ST) network for unique *Brugia* sequences based on location (i.e. country) and host species was created using *cox1* sequences. For this, selected sequences were aligned in Geneious Prime® 2023.2.1 using the MAFFT algorithm and exported as a NEXUS file. The information on the location and the host species were manually included in a ‘trait block’ and the Minimum Spanning Network (at epsilon = 0) was constructed using PopART version 1.7.^25^

The phylogenetic trees and ST network were edited in Affinity Designer 2 (Serif Europe Ltd.) Version 2.2.1 to improve clarity and aesthetics.

### Statistical analysis

The mean 95% and standard deviation (SD) of the assessed individuals were calculated using DescTools package^26^ in R studio (2026.01.0 Build 392). This cross-sectional study followed STROBE (strengthening the reporting of observational studies in epidemiology) guidelines.^27^

### Ethics statement

Use of these samples in this study was approved by the Ethics Review Committee of the Postgraduate Institute of Medicine, University of Colombo, Sri Lanka (ID: ERC/PGIM/2023/133). Informed consent was obtained from adults, and assent was provided by the parents of the children sampled.

### Role of the funding source

The funders of this study had no role in study design, data collection, data analysis, data interpretation, or writing of the report.

## Results

### Demographics and night blood survey surveillance data

A total of 1,855,165 NBS were examined from 2019 through 2023, of which 52 smears had microfilaria morphologically resembling those of *Brugia* spp. (Table 1). Ten whole blood samples from these positive individuals were collected from regions indicated in Supplementary Figure S1 with one additional sample with microfilarial morphology consistent with *W. bancrofti* (total whole blood samples collected: n = 11). Most *Brugia*-positive individuals were males (n = 9) and aged between 1 and 60 years (mean = 11.3, SD ± 16.4). None of these individuals had clinically apparent LF.

**Table 1:** Night blood survey (NBS) surveillance data from regions endemic for lymphatic filariasis in Sri Lanka.

District	Province	NBS samples examined	**NBS samples positive for *Wuchereria bancrofti* mff**	NBS samples positive for *Brugia* spp. mff	Whole blood samples collected and subject to molecular analyses
Kurunegala	North-western	207,826	3	0	0
Puttalam	North-western	92,071	0	10	5
Galle	Southern	321,025	66	11	4
Hambanthota	Southern	58,029	11	1	0
Matara	Southern	313,563	42	2	1
Colombo	Western	289,866	15	8	0
Gampaha	Western	256,922	7	3	1
Kalutara	Western	315,863	9	17	0
**Total**		**1,855,165**	**153**	**52**	**11**

The number of individuals detected with sheathed microfilariae (mff) resembling *Brugia* spp. or *Wuchereria bancrofti* and the number of whole blood samples collected and subjected to molecular analyses for this study are also indicated.

### Filarial worm metabarcoding

From the eleven whole blood samples collected a total of 1,965,339 raw reads were generated, processed and filtered by NanoCLUST into 1,200,341 polished and utilisable reads. The ratio of passed bases (those that met a Q-score of ≥9) to failed bases, was 8.19 Gb:2.09 Gb with a total run duration of 48 h. For the *cox1* gene sequences pre- and post-filtering sample read counts varied substantially with the mean and SE across all biological samples pre-filtering being 187,332 ± 28,557 and post-filtering being 105,664 ± 20,178. Read counts were not normally distributed hence the average count is better represented by the median which was 160,646 for read counts pre-filtering with a range of 24,546–351,311 and 78,451 for read counts post-filtering with a range of 12,964–206,171. For the rDNA region the mean read counts and SE for the biological samples pre-filtering was 30,086 ± 4493 and post-filtering was 28,574 ± 4405. Read counts medians were 34,699 pre-filtering with a range of 5822–59,216 and 32,686 post-filtering with a range of 4950–56,453.

Across the metabarcoding dataset generated only reads classified as filarial worm *cox1* and rDNA sequences was extracted and used for downstream analysis. Non-filarial worm sequences produced by off-target amplification as well as chimeric reads were filtered and removed. When using the *cox1* gene for taxonomic classification only the filarioid pathogens *Brugia* SL genotype (Table 1) and two *W. bancrofti* were identified from clinical samples, with no co-infections identified. The *Brugia* sequences detected in human clinical samples were compared to the NCBI GenBank database and were found to be most similar (99.4–100% nucleotide identity with 100% query cover) to isolates identified in canines from Sri Lanka and Tamil Nadu (accession numbers OR019674.1 and MN564741.1). The taxonomic classifications made using the rDNA region supported those attained when using the *cox1* gene. The same samples were found positive to *Brugia*, however using the rDNA region two comparable top hits were obtained when compared to the NCBI GenBank database *B. pahangi* (96.8%–97.8% identical with 65–70% query cover) and *B. malayi* (95.9%–96.6% identical with 65–70% query cover) see Table 2. The comparably low level of sequence identity between our rDNA sequences when compared to reference sequences from GenBank meant that a species level classification could not be obtained when using this target locus. Such results indicate that the rDNA sequences obtained from the human *Brugia* infections in this study pertain to a *Brugia* species for which no publicly available rDNA sequence data were available. The *W. bancrofti* taxonomic classifications identified when using the rDNA region were consistent with those obtained using the *cox1* gene (Table 2).

**Table 2:** Demographic data and results of the metabarcoding assays targeting the filarial worm *cox1* gene and ribosomal DNA (rDNA) regions from eleven human clinical samples identified as positive for *Brugia* spp. or *Wuchereriabancrofti* microfilariae.

ID	**Sex**	**Age**	District	Microscopic identification^a^	*cox1*									rDNA						
					NCBI reference sequence accession	Species^a^	Identity (%)	Query cover (%)	Length (bp)	NCBI accession	Total reads	Filarial worm reads (%)	NCBI reference sequence accession	Species^a^	Identity (%)	Query cover (%)	Length	NCBI accession	Total reads	Filarial worm reads (%)
1	F	1.75	Gampaha	*Brugia* spp.	AP017705.1	Wb	99.6	100	669	PX933077	126,483	86,369 (68%)	EU272178.1	Wb	100	42	1643	PX956650	34,699	32,686 (94%)
2	M	2.5	Puttlam	*Brugia* spp.	PP931128.1	*Brugia* SL	100	100	659	PX933085	324,419	204,532 (63%)	EU373644.1 & EU373624.1	Bp & Bm	96.8 & 96.3^b^	70 & 70	1581	PX956651	36,183	35,042 (97%)
3	M	4	Puttlam	*Brugia* spp.	PP931128.1	*Brugia* SL	99.9	100	669	PX933080	207,053	135,977 (66%)	EU373646.1 & EU373624.1	Bp & Bm	96.9 & 96.2^b^	65 & 65	1683	PV646250	59,216	55,848 (94%)
4	M	60	Kalutara	Wb	AP017705.1	Wb	99.5	99	687	PX933078	253,134	171,274 (68%)	EU272178.1	Wb	99.9	41	1676	PX956643	18,675	18,128 (97%)
5	M	15	Galle	*Brugia* spp.	PP931128.1	*Brugia* SL	99.7	100	661	PX933081	24,546	17,055 (69%)	EU373646.1 & EU373624.1	Bp & Bm	96.8 & 96.6^b^	66 & 66	1674	PX956649	5822	4914 (84%)
6	M	19	Galle	*Brugia* spp.	PP931128.1	*Brugia* SL	100	100	675	PX933082	70,223	45,235 (64%)	EU373649.1 & EU373624.1	Bp & Bm	97.8 & 95.9^b^	67 & 66	1659	PX956648	37,683	36,090 (96%)
7	F	12	Galle	*Brugia* spp.	PP931128.1	*Brugia* SL	100	100	679	PX933079	309,477	192,279 (62%)	EU373646.1 & EU373624.1	Bp & Bm	96.9 & 96.5^b^	66 & 66	1675	PX956644	19,749	19,008 (96%)
8	M	5	Galle	*Brugia* spp.	PP931128.1	*Brugia* SL	100	100	666	PP931128	351,311	200,717 (57%)	EU373638.1 & EU373624.1	Bp & Bm	97.2 & 96.3^b^	66 & 66	1666	PV646251	8087	5310 (66%)
9	M	8	Matara	*Brugia* spp.	PP931128.1	*Brugia* SL	100	100	671	PX933083	139,938	66,219 (47%)	EU373649.1 & EU373624.1	Bp & Bm	96.8 & 96.2^b^	66 & 66	1674	PX956645	32,846	31,863 (97%)
10	M	6	Galle	*Brugia* spp.	PP931128.1	*Brugia* SL	100	100	689	PP931129	162,537	12,199 (8%)	EU373649.1 & EU373624.1	Bp & Bm	96.7 & 96.4^b^	68 & 68	1632	PX956647	37,854	34,998 (92%)
11	M	1.5	Puttlam	*Brugia* spp.	PP931128.1	*Brugia* SL	100	100	686	PX933084	120,102	54,224 (45%)	EU373646.1 & EU373624.1	Bp & Bm	96.7 & 96.4^b^	65 & 65	1687	PX956646	40,127	38,878 (97%)

Identity and query cover scores as well as the NCBI reference sequence accessions they pertain to were obtained by classifying metabarcoding sequences against NCBI's GenBank using blastn. Microscopy-based identification could only be conducted down to genus level for some filarioids. The sequences generated in this study are shown in the NCBI accession column.

a Wb = *Wuchereria bancrofti*; Bm = *Brugia malayi*; Bp = *Brugia pahangi*; *Brugia* SL = *Brugia* Sri Lanka genotype.

b Percentage identity values are low for the *Brugia* taxon identified from the present study due to our rDNA sequences not being similar to any publicly available rDNA data within NCBI's GenBank.

Unique *cox1* and rDNA sequences generated in the present study were uploaded to NCBI's GenBank and given the accession numbers PP931127–PP931129 and PX933077–PX933085 for *cox1* sequences and PV646248–PV646251 and PX956643–PX956651 for rDNA sequences.

### Phylogenetic analyses and sequence type network

**Fig. 2:**
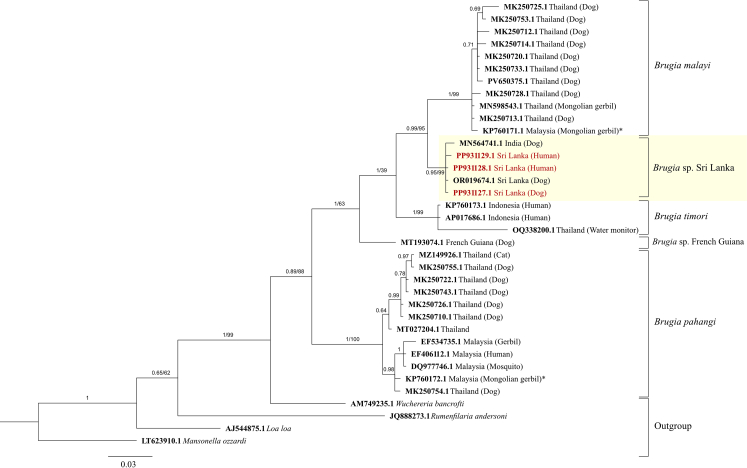
**Bayesian and neighbour-joining phylogenetic analyses using a 417 bp portion of the mitochondrial *cox1* gene of *Brugia* species identified here and in previous studies**. All unique *Brugia* sequences available in NCBI's GenBank database as of 23.02.24, and human and canine *Brugia* sequences obtained from this study (red text) and others were compared. Yellow highlighting shows the *Brugia* Sri Lanka genotype clade. Tree branches are labelled with posterior probability values/bootstrap support (where available) indicating statistical confidence that a specific branch or clade is correct.

**Fig. 3:**
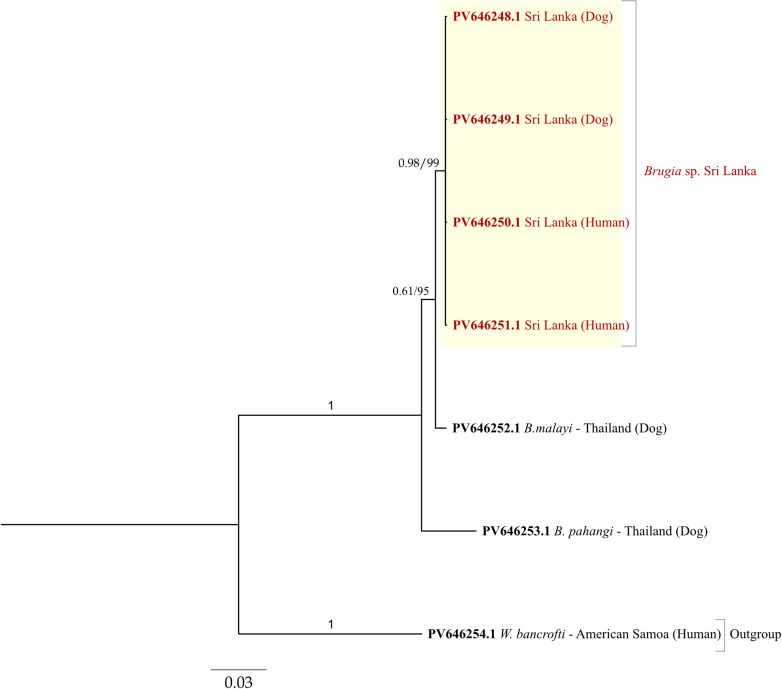
**Bayesian and neighbour-joining phylogenetic analyses using a 1270 bp portion of the nuclear ribosomal RNA gene of different *Brugia* species**. Sequences were generated using a rDNA targeting metabarcoding assay from known samples of *Wuchereria bancrofti, Brugia malayi, Brugia pahangi*, and dogs previously identified as positive for the *Brugia* Sri Lanka genotype, along with human samples from the present study. Yellow highlighting shows the *Brugia* Sri Lanka genotype clade, whilst red text identifies *Brugia* Sri Lanka genotype sequences generated in the present study. Tree branches are labelled with posterior probability values/bootstrap support (where available) indicating statistical confidence that a specific branch or clade is correct.

A total of 49 *Brugia* mitochondrial *cox1* sequences were downloaded from the GenBank database, along with an additional *B. malayi* positive control that was sequenced using the same protocol applied in this study. These sequences, along with the *Brugia* sequences from dogs from a previous study^14^ and humans in this study, yielded 23 STs. The closest genetic relationship of *Brugia* sequences from humans in this study was to *Brugia* SL genotype isolated from dogs in Sri Lanka (accession number **OR019674**), with a posterior probability of 0.95 in BI and 99% bootstrap support in NJ analysis (Fig. 2). Hence, this clade was distinct from the *B. malayi* clade (Fig. 2). Phylogenetic inference based on a 1270 bp sequence from rDNA sequences was consistent with the results obtained from the mitochondrial *cox1* gene (Fig. 3).

**Fig. 4:**
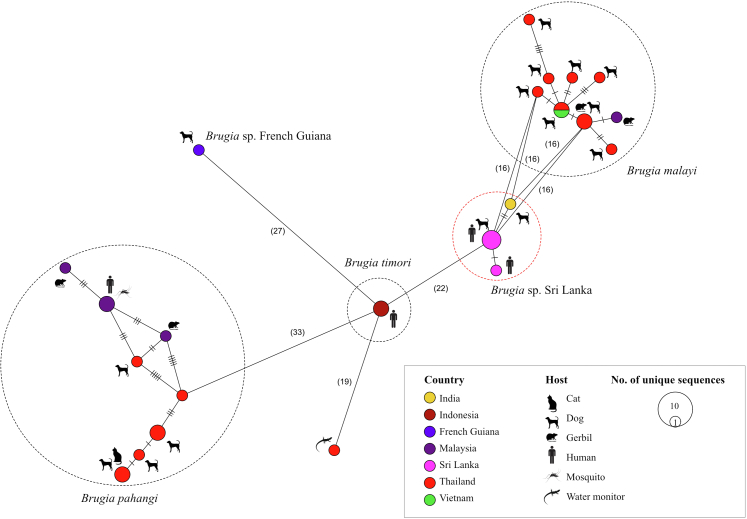
**Minimum Spanning Network (at epsilon = 0) of publicly available sequences and unique mitochondrial *cox1* sequence types for *Brugia* in NCBI's GenBank database as of 23.02.24, showing both their inter- and intra-specific genetic variability according to host species and country**. Number of mutations if ≤ 5 is indicated by hatch markings and those with >5 are indicated inside parentheses.

For MS-ST network construction, 31 unique sequences were identified when both host species and location were considered. In this network, *Brugia* SL clustered separately from *B. malayi*, with 16–22 SNP differences across a 417 bp region of the *cox1* gene (Fig. 4). The *Brugia* STs from dogs and humans in Sri Lanka clustered within the same node (Fig. 4). Additionally, this was the only *Brugia* ST identified in both humans and other mammalian hosts, based on NCBI GenBank data available as of 23.02.24 (Fig. 4).

## Discussion

Here, we provide robust evidence that dogs serve as reservoir hosts for the human-infecting *Brugia* SL genotype during the post–validation surveillance for LF in Sri Lanka. The initial detection of subperiodic *Brugia* infections in humans during post-validation surveillance for LF provided an early indication of a potential zoonotic origin.^5^, ^6^, ^7^, ^8^ Furthermore, a recent investigation of canine filariases reported a notably high prevalence of the *Brugia* sp. SL genotype in provinces where human LF remains endemic.^14^ Nevertheless, at that time, no substantive evidence supported the hypothesis that dogs served as reservoirs for re-emergent brugian filarial infections in humans.^12^^,^^14^

Previous investigations of *Brugia* spp. based on rDNA sequence analyses of samples from dogs in Sri Lanka by Mallawarachchi (2017, 2018)^12^^,^^28^ and Rathnayake (2022)^13^ identified *B. malayi* as the only *Brugia* species present, although genetic divergence from *B. malayi* was noted. The recent delineation of the *Brugia* SL genotype suggests that the *B. malayi* previously reported in dogs in Sri Lanka^12^^,^^13^^,^^28^ most probably represents this genotype. Notably, before these investigations, the only *Brugia* species described in Sri Lankan dogs was *B. ceylonensis*.^11^ However, since its initial description in the 1960s, no further detections have been reported, likely reflecting the historical lack of genetic data and the morphological similarities of *B. ceylonensis* microfilariae from those of *B. malayi*^11^ and *Brugia* SL.^14^ Molecular characterisation of adult *Brugia* SL specimens will be crucial to determine whether this genotype corresponds to the previously described *B. ceylonensis*.

In this study, using a novel metabarcoding platform targeting both nuclear and mitochondrial genomic regions, we provide substantial evidence that dogs act as reservoir hosts for re-emergent brugian infections in humans. Phylogenetic and sequence network analyses of *cox1* gene sequences from *Brugia* isolated from microfilaraemic human and canine blood samples in Sri Lanka, compared with reference sequences from other regions, demonstrated close genetic identity. These findings were further validated using a second nuclear marker encompassing the 18*S* *rRNA* gene, the *ITS*-1 region, the 5.8 rRNA gene, the *ITS*-2 regions, and the 5′ end of the 28*S rRNA* gene. Although the *cox1* gene is widely used for filarial identification due to the abundance of reference data in public databases, rDNA sequences remain underrepresented.^29^ Moreover, many previously generated rDNA sequences are short (∼600 bp) and contain highly conserved regions, limiting their ability to resolve species-level taxonomy, as likely occurred in earlier misclassification of *B. malayi* in Sri Lanka in animals and humans.^12^^,^^13^^,^^28^ Our study's employment of a secondary rDNA locus for taxonomic identification of filarioids underscores a systemic limitation of this target for diagnostic use, i.e. that there is a dearth of filarial worm rDNA regions in publicly available databases, particularly long-read sequences (>1000 bp). This hampers the use of rDNA as a potentially valuable barcoding locus and underscores the need for greater efforts to increase filarioid rDNA representation in databases, ideally through the inclusion of sequences that encompass full-length rDNA sequences. When such databases have adequate representation of filarioid rDNA sequences then this locus may prove to be an ideal alternative barcoding target for filarial worm classification and will lead to greater classification consistencies in circumstances where multiple barcoding targets are used.

The detection of a zoonotic *Brugia* species in dogs is of critical public health importance, given the ubiquity of free-roaming and community-owned dogs in Sri Lanka. These animals often have limited access to veterinary care,^30^ remain untreated, and thus serve as ideal reservoirs for zoonotic filarial infections. Moreover, *Brugia* infections in dogs are typically subclinical,^14^ allowing the persistence of a large, undiagnosed reservoir population. Previous studies in Sri Lanka have also identified the presence of an additional zoonotic filarial worm in over 27% of pet dogs in the country; *Dirofilaria asiatica*.^14^^,^^31^ This recently characterised filarioid causes limited-to-no clinical signs in dogs but can generate harmful zoonotic infections in people through the formation of painful subcutaneous masses or subconjunctival nodules.^14^^,^^31^ Importantly, given that free-roaming dogs spend most of their time outdoors, they are frequently exposed to mosquito bites, further promoting transmission and perpetuation of the life cycles of both *Brugia* SL genotype and *D. asiatica*. The presence of such a widespread, untreated reservoir that closely coexists with humans poses a substantial risk to the long-term success of LF elimination. Although Sri Lanka was declared free of LF as a public health problem in 2016,^4^ widespread canine infection coupled with the cessation of mass drug administration (MDA) in humans could drive infection prevalence above the WHO threshold of <1%.

Current routine surveillance for LF in Sri Lanka relies primarily on thick blood smears (TBS), a method with low sensitivity for detecting microfilariae.^32^ In a previous study, the Modified Knott's Test detected only 18% of infections in dogs, whereas PCR-based methods identified nearly 40%.^14^ Thus, reliance on TBS alone probably underestimates infection prevalence in both humans and animals, meaning that the uptake of more sensitive molecular methods such as PCR, should be considered, particularly in low parasite prevalence contexts. Furthermore, TBS can detect only patent infections, and its specificity depends on observer expertise, as both *W. bancrofti* and *Brugia* spp. have sheathed microfilariae. In this study, one sample initially identified as *Brugia* spp. on TBS was later confirmed molecularly as *W. bancrofti*, highlighting the limitations of microscopy-based diagnosis. Furthermore, given that zoonotic brugian filariasis generally exhibits subperiodic periodicity, the identification of cases through NBS implies that infection prevalence in this population in Sri Lanka is likely higher than that reported.

Given these findings, immediate action is needed to prevent further spreading of zoonotic *Brugia* transmission. A coordinated One Health approach that integrates veterinarians, public health professionals, and community stakeholders will be vital for effective and sustained national LF elimination efforts. Such an intersectoral approach could follow methods akin to those adopted in Thailand, which encompass widespread LF surveillance in humans alongside testing of reservoir species such as cats.^33^ Furthermore, effective post-validation surveillance for LF can be strengthened through continued molecular xenomonitoring of mosquito vectors, as has previosuly implemented in Sri Lanka.^34^ Analysis of countries that have achieved the best LF elimination outcomes has shown that sustained post-validation surveillance, enabled through long-term institutional and governmental commitment that leverages pre-existing health infrastructure, is key.^33^, ^34^, ^35^ Important next steps should now focus on characterising the vector for this newly recognised *Brugia* species, e.g. through xenomonitoring, which will provide crucial insights into how this parasite can best be controlled.^34^ Moreover, thorough investigations into whether other animals are permissive hosts for *Brugia* SL genotype as well as this parasite's responsiveness to chemopreventives and LF treatment will be essential.

A recent study reported low awareness among veterinarians and pet owners on zoonotic filarial infections in Sri Lanka.^36^ Enhancing education among the public, veterinary practitioners, and health authorities about zoonotic filariasis is therefore critical. Future studies should focus on identifying the mosquito vectors responsible for transmission of the *Brugia* SL genotype, which will inform targeted vector control strategies and strengthen surveillance through xenomonitoring. Notably, this genotype has also been identified in a dog in India,^37^ underscoring the need for molecular-based spatial mapping to understand its distribution and potential impact on regional LF control programmes across the Asia–Pacific region.

This study provides molecular evidence of a zoonotic *Brugia* closely related to *B. malayi* infecting humans, with dogs serving as reservoir hosts in Sri Lanka. Due to the low sensitivity of microscopy-based screening for filarioid detection our findings likely represent an underestimation of human cases of this parasite in Sri Lanka. Crucially, these findings highlight the need for a One Health approach to control zoonotic filarial infections nationwide, particularly given the widespread canine reservoir population in Sri Lanka. Timely and sustained surveillance using sensitive molecular diagnostic methods remains essential, as anthroponotic and zoonotic microfilariae are morphologically indistinguishable and infections often remain clinically silent for years before causing disease.

## Contributors

IEG conceptualisation, resources, investigation, methodology, project administration and writing of the original draft and editing. LGH data generation, interpretation and curation, formal analysis, methodology, validation, software, and writing of the original draft and editing. UA investigation, data curation, data interpretation, formal analysis, methodology, software, project administration and writing of the original draft and editing. LL methodology, data collection and interpretation and writing—review & editing. NS data collection and interpretation and writing—review & editing. MV—resources, supervision and writing—review & editing. VC conceptualisation, data interpretation, methodology, resources, supervision, project administration and writing—review & editing. UA, LGH and VC accessed and verified the underlying data. All authors had full access to the data accrued through this study and accept the final responsibility for the decision to submit for publication.

## Data sharing statement

All nanopore sequencing data produced in this study are available from the NCBI BioProject database BioProjectID: PRJNA1125935. Unique *cox1* and rDNA sequences generated in the present study were given NCBI accession numbers PP931127–PP931129 and PX933077–PX933085 as well as PV646248–PV646251 and PX956643–PX956651.

## Editor's note

The Lancet Group takes a neutral position with respect to territorial claims in published maps and institutional affiliations.

## Declaration of interests

The authors declare no competing interests.
