# Supplementary material for: Emergence of a novel zoonotic brugian filarial infection during post-validation surveillance for lymphatic filariasis in Sri Lanka
Source: Lancet Reg Health Southeast Asia. 2026 Mar 4;46:100735. doi: 10.1016/j.lansea.2026.100735 (PMC13005133; doi:10.1016/j.lansea.2026.100735)
Supplement: Supplementary Figure S1 [file mmc1.docx]

**
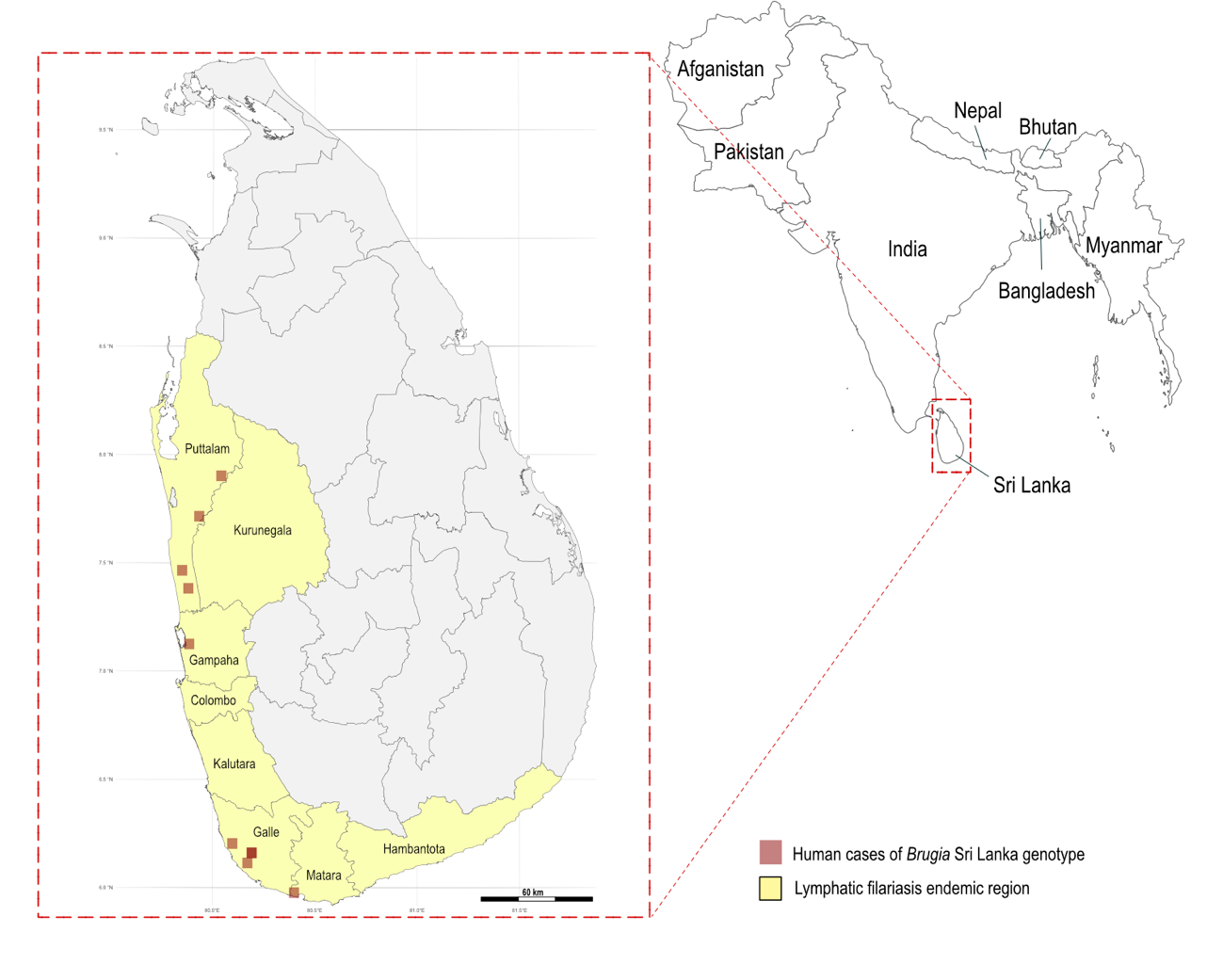
**

**Supplementary figure 1. Geographic map of Sri Lanka with districts endemic for lymphatic filariasis where active surveillance occurred highlighted in yellow**. Locations of individuals molecularly identified with *Brugia* Sri Lanka genotype microfilariae identified using night blood survey are shown in light red squares. Map created using ‘ceylon’ package in R^2^ and improved for clarity using Affinity Designer v 2.5.6.
